# Prognostic implications of alpha-fetoprotein and C-reactive protein elevation in hepatocellular carcinoma following resection (PACE): a large cohort study of 2770 patients

**DOI:** 10.1186/s12885-023-11693-6

**Published:** 2023-12-05

**Authors:** Kong-Ying Lin, Qing-Jing Chen, Shi-Chuan Tang, Zhi-Wen Lin, Jian-Xi Zhang, Si-Ming Zheng, Yun-Tong Li, Xian-Ming Wang, Qiang Lu, Jun Fu, Luo-Bin Guo, Li-Fang Zheng, Peng-Hui You, Meng-Meng Wu, Ke-Can Lin, Wei-Ping Zhou, Tian Yang, Yong-Yi Zeng

**Affiliations:** 1 Department of Hepatopancreatobiliary Surgery, First Affiliated Hospital of Fujian Medical University, Fuzhou, 350000 China; 2 Department of Hepatopancreatobiliary Surgery, Mengchao Hepatobiliary Hospital of Fujian Medical University, Fuzhou, 350000 China; 3 Department of Hepatobiliary Surgery, Xiamen Hospital, Beijing University of Chinese Medicine, Xiamen, 361000 China; 4 Department of Hepatopancreatobiliary Surgery, First Affiliated Hospital of Ningbo University, Ningbo, 315000 China; 5 Department of Hepatobiliary Surgery, Zhongshan Hospital of Xiamen University, Xiamen, 361000 China; 6 Department of General Surgery, First Affiliated Hospital of Shandong First Medical University, Shandong, 250014 China; 7 Department of Hepatopancreatobiliary Surgery, Third Hospital of Zhangzhou, Zhangzhou, 363000 China; 8 Biobank in Mengchao Hepatobiliary Hospital of Fujian Medical University, Fuzhou, 350000 China; 9 Department of Hepatobiliary Surgery, Eastern Hepatobiliary Surgery Hospital, Navy Medical University (Second Military Medical University), Shanghai, 200000 China; 10 Liver Disease Research Center of Fujian Province, Fuzhou, 350000 China; 11 Department of Hepatopancreatobiliary Surgery, Mengchao Hepatobiliary Hospital of Fujian Medical University, Xihong Road 312, Fuzhou, 350025 China

**Keywords:** Hepatocellular carcinoma, Resection, Alpha-fetoprotein, C-reactive protein, Prognosis

## Abstract

**Background:**

Routine clinical staging for hepatocellular carcinoma (HCC) incorporates liver function, general health, and tumor morphology. Further refinement of prognostic assessments and treatment decisions may benefit from the inclusion of tumor biological marker alpha-fetoprotein (AFP) and systemic inflammation indicator C-reactive protein (CRP).

**Methods:**

Data from a multicenter cohort of 2770 HCC patients undergoing hepatectomy were analyzed. We developed the PACE risk score (Prognostic implications of AFP and CRP Elevation) after initially assessing preoperative AFP and CRP’s prognostic value. Subgroup analyzes were performed in BCLC cohorts A and B using multivariable Cox analysis to evaluate the prognostic stratification ability of the PACE risk score and its complementary utility for BCLC staging.

**Results:**

Preoperative AFP ≥ 400ng/mL and CRP ≥ 10 mg/L emerged as independent predictors of poorer prognosis in HCC patients who underwent hepatectomy, leading to the creation of the PACE risk score. PACE risk score stratified patients into low, intermediate, and high-risk groups with cumulative 5-year overall (OS) and recurrence-free survival (RFS) rates of 59.6%/44.9%, 43.9%/38.4%, and 20.6%/18.0% respectively (all ***P < 0.001***). Increased PACE risk scores correlated significantly with early recurrence and extrahepatic metastases frequency (all ***P < 0.001***). The multivariable analysis identified intermediate and high-risk PACE scores as independently correlating with poor postoperative OS and RFS. Furthermore, the PACE risk score proficiently stratified the prognosis of BCLC stages A and B patients, with multivariable analyses demonstrating it as an independent prognostic determinant for both stages.

**Conclusion:**

The PACE risk score serves as an effective tool for postoperative risk stratification, potentially supplementing the BCLC staging system.

**Supplementary Information:**

The online version contains supplementary material available at 10.1186/s12885-023-11693-6.

## Introduction

Hepatocellular carcinoma (HCC) continues to be one of the most pervasive malignancies worldwide, with both disease burden and mortality rates escalating [1, 2]. Liver resection emerges as a pivotal, potentially curative approach for HCC treatment, proving particularly effective for those diagnosed at very early or early stages [3, 4]. Nevertheless, due to the complex biological characteristics of the tumor and individual patient differences, the prognosis can vary substantially even among those who have undergone liver resection [5–7].

The prognosis of HCC depends significantly on the staging of the disease and suitable treatment options [8, 9]. Clinical staging systems exemplified by the Barcelona Clinic Liver Cancer (BCLC) algorithm are utilized for prognosis evaluation and therapeutic recommendations [9]. Despite their pivotal role in HCC treatment decisions, these staging systems have limitations. They often solely account for tumor morphological characteristics and patient baseline conditions, thereby offering limited performance in refining patient prognosis and guiding treatment decisions. It’s not unusual to see patients within the same stage, who have received the same guideline-recommended treatment, exhibit markedly different clinical outcomes [7, 10].

In recent times, an increasing body of evidence has shown that the prognosis of HCC is associated not only with the tumor’s morphological features but also with its biological behavior and the host’s systemic inflammatory response [11–15]. Hematological parameters, as effective indicators of the host’s systemic inflammatory response and tumor biology, have gained wide acceptance. Among these, C-reactive protein (CRP) is a marker of systemic inflammation, and it has been proven to have a strong correlation with the prognosis of numerous tumors [16–18]. Similarly, alpha-fetoprotein (AFP), an HCC-specific biomarker, is considered to reflect the tumor’s biological behavior and clinical prognosis [19, 20]. In consideration of these factors, it is plausible that including hematological parameters like CRP and AFP, indicators of systemic inflammation and HCC biology respectively, in clinical staging evaluations may offer substantial aid in enhancing patient prognostic assessment and treatment allocation.

To this end, we conducted a large-scale retrospective study involving a multicenter cohort of 2770 patients, with the aim of exploring the value of CRP and AFP in the prognostic evaluation of HCC after resection, and constructed a **PACE** risk score (Prognostic implications of AFP and CRP Elevation), with the goal of providing clinicians with additional information on tumor biological aggressiveness and systemic inflammatory response. Our results show that the PACE score, when used in combination with the tumor morphology-based BCLC staging system, has the potential to provide clinicians with more accurate and personalized prognostic assessment and treatment recommendations before surgery, thereby enhancing assessment of suitability for liver resection, postoperative management, and improving the outcome of liver resection.

## Methods

### Study population

A retrospective review was conducted on consecutive patients with HCC who underwent hepatectomy as a curative-intent therapy, between April 2009 and April 2019 at six hospitals in China: Mengchao Hepatobiliary Hospital of Fujian Medical University, Eastern Hepatobiliary Surgery Hospital of Naval Medical University, Zhongshan Hospital affiliated to Xiamen University, the First Affiliated Hospital of Shandong First Medical University, the First affiliated Hospital of Ningbo University, and the Third Hospital of Zhangzhou. HCC diagnoses were confirmed histopathologically from resected specimens. The study’s inclusion criteria were: (1) patients who underwent R0 resection, which is characterized by complete tumor removal with pathologically confirmed absence of tumor cells at the surgical margin; (2) absence of macrovascular invasion and extrahepatic metastasis; (3) availability of preoperative serum AFP and CRP data (collected within one week prior to hepatectomy). Patients were excluded based on the following criteria: (1) recurrent or mixed HCC; (2) clinical evidence of preoperative infection; (3) prior anticancer treatments; (4) palliative resection (R1/R2); (5) emergency hepatectomy due to rupture HCC; (6) insufficient clinical information or missing follow-up data. This retrospective study was approved by the institutional review boards of each medical center and conducted according to the ethical guidelines of the 1975 Declaration of Helsinki.

### Clinicopathologic and operational variables

Patients’ clinicopathologic variables included age, gender, hypertension, diabetes, liver cirrhosis, etiology of liver disease, liver function status, platelet count, total bilirubin, albumin, AFP, CRP, the number of tumors, tumor diameter, satellite nodules, microvascular invasion, degree of tumor cell differentiation, tumor capsule, and BCLC staging. All laboratory indices were based on the most recent tests within one week before hepatectomy. BCLC stage 0 was defined as a single lesion not larger than 2 cm, stage A as a single lesion larger than 2 cm, or multiple lesions numbering 2–3 with the largest tumor not larger than 3 cm, and stage B as more than three tumors or 2–3 tumors with any of them larger than 3 cm in diameter [8]. Operational variables included the extent of hepatectomy, type of hepatectomy, intraoperative blood transfusion, volume of intraoperative blood loss, and resection margin status. The extent of hepatectomy was divided into major (removal of three or more Couinaud segments) and minor (removal of fewer than three Couinaud segments). Types of hepatectomy were divided into anatomical (as per the Brisbane 2000 nomenclature of liver anatomy and resections) and non-anatomical, which includes limited hepatectomy or wedge resection [21].

### Follow-up and study endpoints

Following discharge, patients were monitored for tumor recurrence at outpatient clinics according to the relatively uniform scheme. For the initial two years post-surgery, visits were every 2–3 months, with 3–6 month intervals thereafter if recurrence was absent. Monitoring involved routine blood tests, liver function tests, tumor markers, and radiological assessments encompassing lung imaging, abdominal ultrasound, or enhanced computed tomography/magnetic resonance imaging. Therapeutic strategies for recurrent cases were selected based on their general health status, liver functional reserve, and tumor burden at recurrence, and include repeat hepatectomy, radiofrequency ablation, trans-arterial regional therapy, systemic therapy, and supportive care.

Data for this study was reviewed on October 13, 2022. The primary endpoints were overall survival (OS) and recurrence-free survival (RFS). OS was defined as the period from the date of hepatectomy to the date of patient death or last follow-up. RFS was defined as the interval from the date of hepatectomy to tumor recurrence, death, or the last follow-up, whichever occurred first.

### Statistics

Continuous variables are presented as mean ± standard deviation and were compared using the Mann-Whitney U-test or Kruskal–Wallis one-way ANOVA as appropriate. Categorical variables are represented as frequency (percentage) and compared using χ2 or Fisher’s exact tests as needed. To enhance clinical applicability, this study used the widely reported cut-off values of 400 ng/mL for AFP and 10 mg/L for CRP [16, 17, 22–26]. Cox proportional hazards regression analysis was used to ascertain the relationship between AFP and AFP with post-hepatectomy outcomes. Variables with a *P* < 0.05 in the univariable analysis were included in the multivariable analysis. Given the similar hazard ratios of AFP and CRP in the multivariable analysis for OS and RFS, we developed a simple PACE risk score (Prognostic implications of AFP and CRP Elevation). To construct the PACE score, we stratified patients into three distinct risk categories based on their preoperative AFP and CRP levels: low-risk (0 points), intermediate-risk (1 point), and high-risk (2 points). The points for each category were determined by the presence of AFP ≥ 400 ng/mL and/or CRP ≥ 10 mg/L, with the higher scores reflecting a greater risk of adverse postoperative outcomes. Specifically, 0 points were given for AFP ≤ 400 ng/mL and CRP ≤ 10 mg/L, 1 point for either AFP ≥ 400 ng/mL or CRP ≥ 10 mg/L, and 2 points for AFP ≥ 400 ng/mL and CRP ≥ 10 mg/L. The range of the PACE score is 0 to 2, with scores assigned from 0 (indicating low risk) to 2 (indicating high risk). The performance of PACE and PACE combined with BCLC staging system was measured using the concordance index (C-index) and calibration curve used the bootstrap with 1000 resamples. In addition, time-dependent receiver operating characteristic curves were plotted. To evaluate the independent prognostic significance of the PACE score from BCLC staging, we examined its influence on the outcomes after hepatectomy within the BCLC A and B stage cohorts using multivariable Cox regression analysis (analysis was not performed for BCLC 0 stage patients due to sample size limitations). We used the Schoenfeld residual test to verify the assumption of proportional hazards in the Cox analysis.

All statistical analyses for this study were conducted using SPSS version 20 and R version 4.1.1.

## Results

### Patients characteristics

In total, 2770 patients were enrolled in the study. Patient baseline characteristics are outlined in Table 1. The mean age of patients was 53.2 ± 11.1 years. The predominant liver disease was hepatitis B virus infection (2358, 85.1%). According to BCLC staging, 159 (5.7%), 2158 (77.9%), and 453 (16.4%) patients were identified as stages 0, A, and B, respectively.

**Table 1:** Baseline clinicopathological characteristics

Variables	Total cohort (N = 2770)	PACE low-risk (N = 1665)	PACE intermediate-risk (N = 958)	PACE high-risk (N = 147)	*P-value*
**Age**, years, Mean (SD)	53.2 (11.1)	54.7 (10.5)	51.3 (11.4)	48.2 (11.7)	< 0.001
**Gender**					
Female	425 (15.3%)	212 (12.7%)	191 (19.9%)	22 (15.0%)	< 0.001
Male	2345 (84.7%)	1453 (87.3%)	767 (80.1%)	125 (85.0%)	
**Diabetes**	259 (9.4%)	189 (11.4%)	61 (6.4%)	9 (6.1%)	< 0.001
**Hypertension**	525 (19.0%)	333 (20.0%)	167 (17.4%)	25 (17.0%)	0.224
**Etiology**					
HBV	2358 (85.1%)	1390 (83.5%)	837 (87.4%)	131 (89.1%)	0.012
HCV	35 (1.3%)	29 (1.7%)	6 (0.6%)	0 (0%)	
Non-B, non-C	370 (13.4%)	241 (14.5%)	114 (11.9%)	15 (10.2%)	
HBV, HCV	7 (0.3%)	5 (0.3%)	1 (0.1%)	1 (0.7%)	
**Child-Pugh class**					
A	2564 (92.6%)	1561 (93.8%)	878 (91.6%)	125 (85.0%)	< 0.001
B	206 (7.4%)	104 (6.2%)	80 (8.4%)	22 (15.0%)	
**BCLC staging system**					
0	159 (5.7%)	132 (7.9%)	27 (2.8%)	0 (0%)	< 0.001
A	2158 (77.9%)	1297 (77.9%)	753 (78.6%)	108 (73.5%)	
B	453 (16.4%)	236 (14.2%)	178 (18.6%)	39 (26.5%)	
**Platelet**, Mean (SD), 10^9^/L	167 (69.8)	155 (61.9)	177 (72.2)	227 (93.7)	< 0.001
**Total bilirubin**, Mean (SD), umol/L	15.1 (11.1)	15.2 (12.2)	15.0 (9.54)	15.0 (6.06)	0.895
**Albumin**, Mean (SD), g/L	41.5 (3.89)	41.8 (3.81)	41.2 (3.91)	39.5 (3.92)	< 0.001
**AFP**, ng/mL					
< 400	1876 (67.7%)	1665 (100%)	211 (22.0%)	0 (0%)	< 0.001
≥ 400	894 (32.3%)	0 (0%)	747 (78.0%)	147 (100%)	
**CRP**, mg/L					
< 10	2412 (87.1%)	1665 (100%)	747 (78.0%)	0 (0%)	< 0.001
≥ 10	358 (12.9%)	0 (0%)	211 (22.0%)	147 (100%)	
**Tumor number**					
Solitary	2236 (80.7%)	1371 (82.3%)	757 (79.0%)	108 (73.5%)	0.008
Multiple	534 (19.3%)	294 (17.7%)	201 (21.0%)	39 (26.5%)	
**Tumor diameter**, Mean (SD), cm	6.01 (3.93)	4.95 (3.11)	7.02 (4.17)	11.4 (4.40)	< 0.001
**Satellite nodules**	1269 (45.8%)	699 (42.0%)	490 (51.1%)	80 (54.4%)	< 0.001
**Tumor differentiation**					
I / II	323 (11.7%)	270 (16.2%)	49 (5.1%)	4 (2.7%)	< 0.001
III / IV	2447 (88.3%)	1395 (83.8%)	909 (94.9%)	143 (97.3%)	
**MVI**	1124 (40.6%)	586 (35.2%)	450 (47.0%)	88 (59.9%)	< 0.001
**Tumor capsule**					
Complete	414 (14.9%)	292 (17.5%)	107 (11.2%)	15 (10.2%)	< 0.001
Incomplete	1862 (67.2%)	1089 (65.4%)	664 (69.3%)	109 (74.1%)	
None	494 (17.8%)	284 (17.1%)	187 (19.5%)	23 (15.6%)	
**Liver cirrhosis**	1743 (62.9%)	1057 (63.5%)	601 (62.7%)	85 (57.8%)	0.391
**Extend of hepatectomy**					
Minor	2268 (81.9%)	1477 (88.7%)	715 (74.6%)	76 (51.7%)	< 0.001
Major	502 (18.1%)	188 (11.3%)	243 (25.4%)	71 (48.3%)	
**Intraoperative blood loss, mL**					
< 800	2625 (94.8%)	1611 (96.8%)	900 (93.9%)	114 (77.6%)	< 0.001
≥ 800	145 (5.2%)	54 (3.2%)	58 (6.1%)	33 (22.4%)	
**Intraoperative blood transfusion**	194 (7.0%)	76 (4.6%)	80 (8.4%)	38 (25.9%)	< 0.001
**Hepatectomy type**					
Non-anatomical	1875 (67.7%)	1137 (68.3%)	650 (67.8%)	88 (59.9%)	0.111
Anatomical	895 (32.3%)	528 (31.7%)	308 (32.2%)	59 (40.1%)	
**Resection margin**					
< 1 cm	1581 (57.1%)	922 (55.4%)	563 (58.8%)	96 (65.3%)	0.028
≥ 1 cm	1189 (42.9%)	743 (44.6%)	395 (41.2%)	51 (34.7%)	

Abbreviations: *PACE*, Prognostic implications of Alpha-fetoprotein and C-reactive protein Elevation; *AFP*, alpha-fetoprotein; *CRP*, C-reactive protein; *HBV*, hepatitis B virus; *HCV*, hepatitis C virus; *PLT*, platelet; *MVI*, microvascular invasion; *BCLC*, Barcelona Clinic Liver Cancer; *SD*, standard deviation

### Prognostic role of AFP and CRP, and construction of PACE risk score

**Fig. 1:**
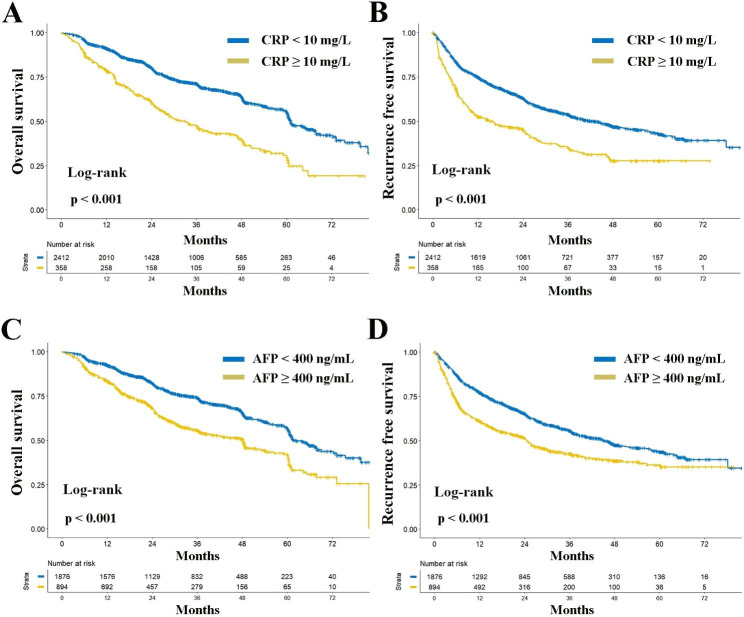
Comparative analysis of overall (**A**) and recurrence-free (**B**) survival between patients with preoperative CRP < 10 mg/L and CRP ≥ 10 mg/L; comparative analysis of overall (**C**) and recurrence-free (**D**) survival between patients with preoperative AFP < 400 ng/mL and AFP ≥ 400 ng/mL. Abbreviations: *CRP*, c-reactive protein; *AFP*, alpha-fetoprotein

The preoperative AFP level was ≥ 400ng/mL in 894 (32.3%) patients and the preoperative CRP level was ≥ 10 mg/L in 358 (12.9%) patients. Comparisons of baseline characteristics between patients with high and low preoperative AFP or CRP levels are detailed in Table S1. Both elevated preoperative AFP and CRP levels are linked to larger tumor diameter, a higher incidence of multifocal disease, microvascular invasion, and more advanced BCLC staging—all of which are recognized indicators of tumor aggressiveness (all ***P < 0.05***, Table S1). Survival analyses revealed a significant association between elevated levels of AFP and CRP and worse postoperative OS and RFS (all ***P < 0.001***, Fig. 1). Multivariable analyses further identified elevated serum CRP and AFP levels as independent prognostic factors for OS and RFS (all ***P < 0.05***, Tables S2 and S3). Based on these findings, we constructed the PACE score according to whether patients met the criteria of AFP ≥ 400ng/mL and/or CRP ≥ 10 mg/L, designating low-risk (neither criteria met), intermediate-risk (either criteria met), and high-risk (both criteria met) groups.

### PACE risk score relation to clinicopathological features and outcomes

Increased PACE risk level correlated with more advanced BCLC staging, larger tumor diameter, higher frequency of multiple nodules, satellite lesions, microvascular invasion, and absence or incompleteness of tumor capsule, along with poorer tumor differentiation (all ***P < 0.05***, Table 1).

**Fig. 2:**
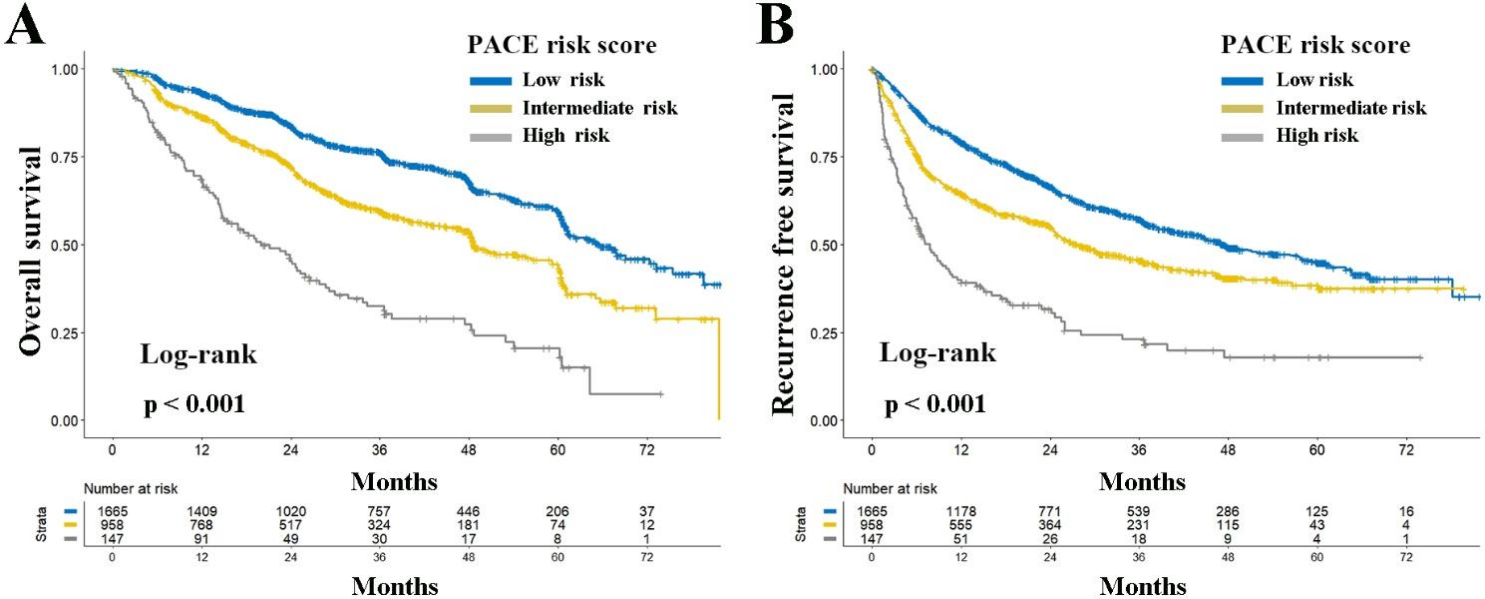
Comparisons of overall (**A**) and recurrence-free (**B**) survival among low, intermediate, and high-risk groups according to PACE risk score in the total cohort. Abbreviations: *PACE*, Prognostic implications of Alpha-fetoprotein and C-reactive protein Elevation

Kaplan-Meier analyses showed significant prognostic differences among PACE risk groups, as increasing PACE risk levels corresponded to a progressive decline in cumulative OS and RFS rates (all ***P < 0.001***, Fig. 2). Additionally, when compared to the low-risk group, both intermediate and high-risk groups showed discouraging recurrence patterns (Table 2). Notably, extrahepatic recurrence occurred more often in intermediate and high-risk groups (low-risk: n = 25, 3.7% vs. intermediate-risk: n = 40, 8.3% vs. high-risk: n = 7, 6.8%; *P < 0.001*, Table 2). Furthermore, high-risk patients demonstrated a higher frequency of early recurrence (within 24 months) (low-risk: n = 502, 73.4% vs. intermediate-risk: n = 403, 82.2% vs. high-risk: n = 93, 90.3%, Table 2). Multivariable analyses indicated that intermediate and high-risk PACE scores were independent risks for both OS (HR: 1.413 and 2.425; 95%CI 1.226–1.628 and 1.891–3.109; all ***P < 0.001***) and RFS (HR: 1.433 and 2.517; 95%CI 1.244–1.650 and 1.964–3.225; all ***P < 0.001***) (Tables 3 and 4).

**Table 2:** Long-term outcomes after resection relative to PACE risk score

Variable	Total	PACE low-risk	PACE intermediate-risk	PACE high-risk	*P*
**Median RFS, months**	37.4 (35.0, 42.2)	47.5 (43.5, 57.3)	27.8 (24.8, 34.3)	7.7 (6.0, 10.8)	**< 0.001** ^*^
1-year RFS	72.0 (70.4, 73.8)	79.1 (77.2, 81.1)	64.4 (61.4, 67.6)	40.0 (32.5, 49.1)	
3-year RFS	51.5 (49.4, 53.6)	57.3 (54.7, 60.0)	45.5 (42.0, 49.1)	23.0 (162, 32.7)	
5-year RFS	41.2 (38.6, 43.9)	44.9 (41.5, 48.5)	38.4 (34.4, 43.0)	18.0 (11.5, 28.3)	
**Recurrence, n, %**	1272 (45.9%)	684 (41.1%)	485 (50.6%)	103 (70.1%)	**< 0.001** ^**#**^
**Recurrence Timing**					
Early recurrence, ≤ 24 months	998 (78.5%)	502 (73.4%)	403 (82.2%)	93 (90.3%)	**< 0.001** ^**#**^
Late recurrence, > 24 months	274 (21.5%)	182 (26.6%)	82 (17.8%)	10 (9.7%)	
**Recurrence pattern**					
Intrahepatic	1052 (82.7%)	592 (86.5%)	376 (77.5%)	84 (81.6%)	**0.001** ^**#**^
Extrahepatic	72 (5.7%)	25 (3.7%)	40 (8.3%)	7 (6.8%)	
Both	148 (11.6%)	67 (9.8%)	69 (14.2%)	12 (11.6%)	
**Death, n, %**	947 (34.2%)	464 (27.9%)	387 (40.4%)	96 (65.3%)	**< 0.001** ^**#**^
**Median OS, months**	60.4 (59.4, 61.1)	65.5 (61.3, 75.4)	48.7 (48.1, 59.2)	19.9 (14.7, 26.6)	**< 0.001** ^*^
1-year OS	89.7 (88.6, 90.9)	93.5 (92.3, 94.7)	86.4 (84.3,88.7)	68.8 (61.6, 76.9)	
3-year OS	68.2 (66.2, 70.1)	76.3 (74.0, 78.6)	59.5 (56.0, 63.1)	32.5 (24.9, 42.4)	
5-year OS	52.1 (49.4, 54.9)	59.6 (56.1, 63.2)	43.9 (39.2, 49.0)	20.6 (13.5, 31.5)	

Abbreviations: *PACE*, Prognostic implications of Alpha-fetoprotein and C-reactive protein Elevation; *RFS*, recurrence-free survival; *OS*, overall survival

*Tested by log-rank test; ^#^Tested by χ2 test

**Table 3:** Univariable and multivariable Cox regression analyses on risk factors of overall survival

Variables	HR comparison	UV HR (95% CI)	UV *P*	MV HR (95% CI)	MV *P*
Age	> 55 vs. ≤55 years	0.867 (0.762–0.986)	0.030	NS	0.981
Gender	Male vs. female	1.024 (0.856–1.225)	0.795		
Diabetes	Present vs. absent	1.061 (0.856–1.316)	0.588		
Hypertension	Present vs. absent	0.963 (0.814–1.140)	0.663		
HBsAg	Positive vs. negative	1.113 (0.922–1.343)	0.266		
HCVAb	Positive vs. negative	0.873 (0.494–1.544)	0.641		
Child-Pugh	B vs. A	1.569 (1.261–1.952)	< 0.001	1.377 (1.101–1.723)	0.005
Cirrhosis	Present vs. absent	0.862 (0.756–0.981)	0.025	NS	0.126
PLT	< 100 vs. ≥100 10^9^/L	1.076 (0.910–1.273)	0.389		
Tumor number	Multiple vs. solitary	2.437 (2.002–2.966)	< 0.001	1.420 (1.209–1.668)	< 0.001
Tumor diameter	≥ 10 vs. <10 cm	1.114 (1.099–1.129)	< 0.001	1.527 (1.283–1.817)	< 0.001
Tumor differentiation	III/IV vs. I/II	2.279 (1.784–2.910)	< 0.001	1.619 (1.253–2.092)	< 0.001
Tumor capsule	Incomplete vs. complete	1.397 (1.148–1.699)	0.001	1.065 (0.869–1.304)	< 0.001
Tumor capsule	None vs. complete	1.986 (1.589–2.482)	< 0.001	1.491 (1.183–1.881)	< 0.001
Satellite nodules	Presence vs. absence	1.861 (1.635–2.118)	< 0.001	1.236 (1.059–1.443)	< 0.001
Intraoperative blood loss	≥ 800 ml vs. <800 ml	2.163 (1.724–2.713)	< 0.001	1.334 (1.043–1.706)	0.021
Anatomical hepatectomy	Yes vs. no	0.837 (0.726–0.964)	0.014	NS	0.511
Resection margin	≥ 1 cm vs. <1 cm	0.602 (0.525–0.689)	< 0.001	0.699 (0.595–0.821)	< 0.001
PACE risk score	intermediate-risk vs. low-risk	1.725 (1.506–1.975)	< 0.001	1.413 (1.226–1.628)	< 0.001
PACE risk score	high-risk vs. low-risk	3.974 (3.187–4.956)	< 0.001	2.425 (1.891–3.109)	< 0.001

Abbreviations: *PACE*, Prognostic implications of Alpha-fetoprotein and C-reactive protein Elevation; *HBV*, hepatitis B virus; *HCV*, hepatitis C virus; *PLT*, platelet; *HR*, hazard ratio; *CI*, confidence interval; *UV* univariable

Schoenfeld residuals test for proportional hazards assumption: met (*P* = 0.102)

**Table 4:** Univariable and multivariable Cox regression analyses on risk factors of recurrence-free survival

Variables	HR comparison	UV HR (95% CI)	UV *P*	MV HR (95% CI)	MV *P*
Age	> 55 vs. ≤55 years	0.964 (0.863–1.076)	0.512		
Gender	Male vs. female	1.044 (0.895–1.218)	0.582		
Diabetes	Present vs. absent	0.989 (0.817–1.197)	0.910		
Hypertension	Present vs. absent	0.887 (0.767–1.026)	0.107		
HBsAg	Positive vs. negative	1.355 (1.142–1.608)	0.001	NS	0.527
HCVAb	Positive vs. negative	0.840 (0.513–1.376)	0.490		
Child-Pugh	B vs. A	1.318 (1.078–1.611)	0.007	1.325 (1.060–1.655)	0.013
Cirrhosis	Present vs. absent	1.078 (0.961–1.210)	0.199		
PLT	< 100 vs. ≥100 10^9^/L	0.981 (0.844–1.140)	0.801		
Tumor number	Multiple vs. solitary	2.211 (1.840–2.656)	< 0.001	1.379 (1.175–1.620)	< 0.001
Tumor diameter	≥ 10 vs. <10 cm	2.163 (1.896–2.468)	< 0.001	1.606 (1.352–1.909)	< 0.001
Tumor differentiation	III/IV vs. I/II	1.399 (1.166–1.678)	< 0.001	1.681 (1.303–2.169)	< 0.001
Tumor capsule	Incomplete vs. complete	1.214 (1.035–1.424)	0.017	NS	0.511
Tumor capsule	None vs. complete	1.428 (1.180–1.728)	< 0.001	1.601 (1.271–2.017)	< 0.001
Satellite nodules	Presence vs. absence	1.396 (1.250–1.560)	< 0.001	1.396 (1.206–1.617)	< 0.001
Intraoperative blood loss	≥ 800 ml vs. <800 ml	1.975 (1.599–2.440)	< 0.001	1.312 (1.026–1.678)	0.031
Anatomical hepatectomy	Yes vs. no	0.771 (0.682–0.872)	< 0.001	NS	0.427
Resection margin	≥ 1 cm vs. <1 cm	0.678 (0.605–0.761)	< 0.001	0.697 (0.592–0.819)	< 0.001
PACE risk score	intermediate-risk vs. low-risk	1.468 (1.306–1.649)	< 0.001	1.433 (1.244–1.650)	< 0.001
PACE risk score	high-risk vs. low-risk	3.065 (2.490–3.774)	< 0.001	2.517 (1.964–3.225)	< 0.001

Abbreviations: *PACE*, Prognostic implications of Alpha-fetoprotein and C-reactive protein Elevation; *HBV*, hepatitis B virus; *HCV*, hepatitis C virus; *PLT*, platelet; *MVI*, microvascular invasion; *HR*, hazard ratio; *CI*, confidence interval; *UV* univariable

Schoenfeld residuals test for proportional hazards assumption: met (*P* = 0.139)

Moreover, we explored the discriminative and calibration capabilities of the PACE score, particularly when combined with the BCLC staging system. As depicted in Table S4, PACE alone provided C-indices of 0.604 for OS and 0.584 for DFS. It is of note that the amalgamation of PACE with BCLC staging improved the predictive accuracy of BCLC staging, with C-indices for OS and DFS at 0.638 and 0.610, respectively. This enhancement was also reflected in the time-dependent ROC curves (Fig. S1), and the calibration curves showed favourable concordance for both PACE and its combination with BCLC staging (Fig. S2).

### Subgroup analysis of BCLC A/B cohort: refinement capability of the PACE risk score

Low, intermediate, and high-risk PACE groups represented 60.1%, 34.9%, and 5% of patients at BCLC stage A, and 52.1%, 39.3%, and 8.6% at BCLC stage B, respectively.

**Fig. 3:**
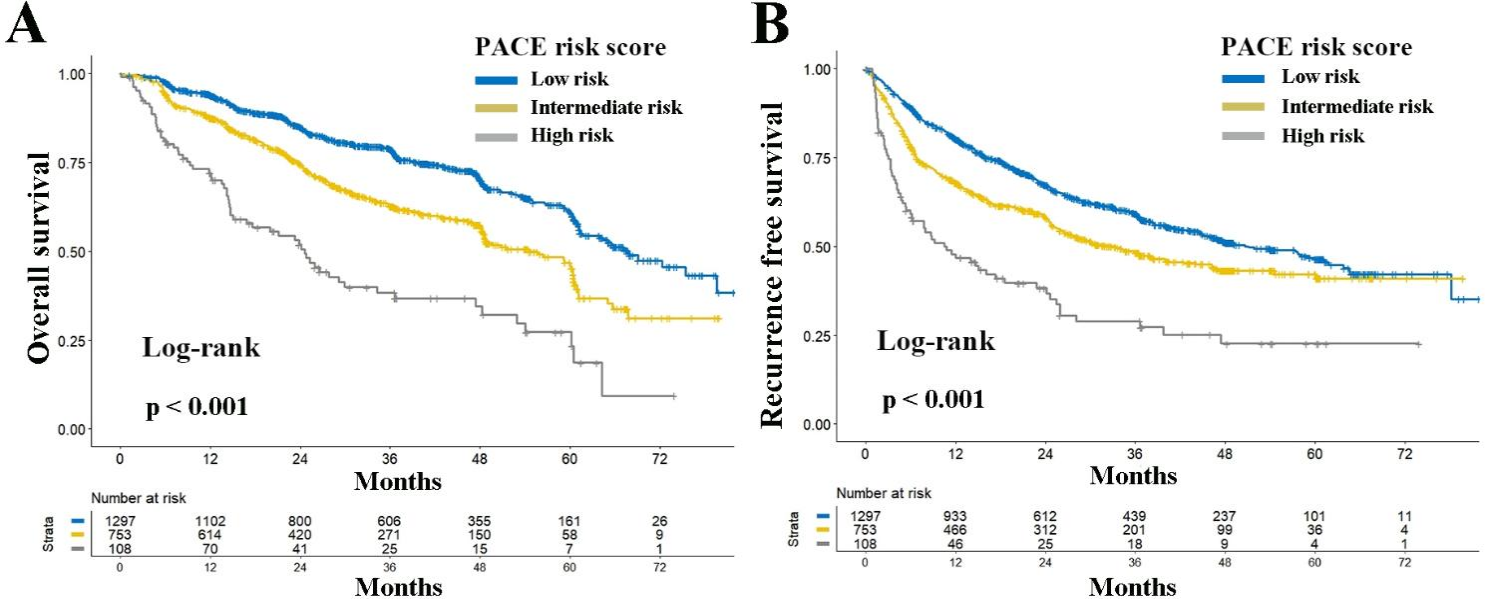
Comparison of overall (**A**) and recurrence-free (**B**) survival among different risk groups according to PACE risk score for patients with BCLC stage A. Abbreviations: *PACE*, Prognostic implications of Alpha-fetoprotein and C-reactive protein Elevation; *BCLC*, Barcelona Clinic Liver Cancer

As per PACE risk status, a gradual deterioration in cumulative OS was noted across the different groups within BCLC stage A (all ***P < 0.001***, Fig. 3A). Median OS and 5-year OS rates for the low, intermediate, and high-risk groups within BCLC stage A were 67.8 months and 61.2%, 54.6 months and 46.1%, and 24.6 months and 27.3% respectively (***P < 0.001***). Multivariable analysis revealed that the intermediate and high-risk PACE categories were independent risk factors for postoperative OS in BCLC stage A patients (HR: 1.448 and 2.468; 95% CI 1.228–1.706 and 1.831–3.327; all ***P < 0.001***, Table S5). Similar patterns were discerned in terms of RFS across different risk groups within BCLC stage A, with multivariable analysis further validating that the intermediate and high-risk PACE statuses were independent risk factors for postoperative RFS in BCLC stage A patients (Fig. 3B and Table S6).

**Fig. 4:**
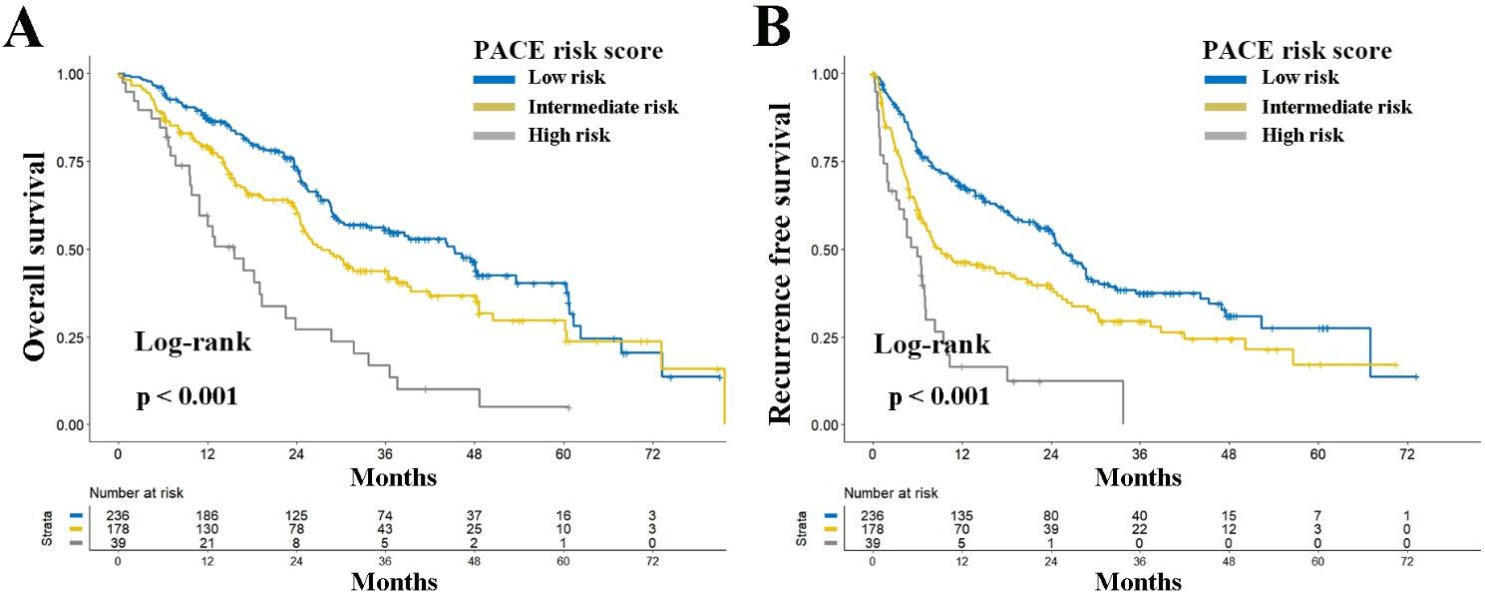
Comparison of overall (**A**) and recurrence-free (**B**) survival among different risk groups according to PACE risk score for patients with BCLC stage B. Abbreviations: *PACE*, Prognostic implications of Alpha-fetoprotein and C-reactive protein Elevation; *BCLC*, Barcelona Clinic Liver Cancer

Similarly, the prognostic significance of the PACE was assessed in the cohort of BCLC stage B. The median OS and 5-year OS rates in BCLC stage B for the low-risk, intermediate-risk, and high-risk groups were 45.3 months and 40.4%, 27.3 months and 29.7%, and 15.6 months and 5.1% respectively (***P < 0.001***, Fig. 4A). Multivariable analysis demonstrated that the PACE risk score is an independent determinant of postoperative OS in patients with BCLC stage B (HR: 1.312 and 2.680; 95% CI 0.974–1.766 and 1.675–4.289; ***P = 0.074*** and ***P < 0.001***, Table S7). Correspondingly, a significant difference was observed in the RFS between different risk groups in BCLC stage B, with multivariable analysis further verifying the PACE risk score as an independent risk factor for postoperative RFS in patients with BCLC stage B (Fig. 4B and Table S8).

## Discussion

Our multicentre, large-cohort study elucidates that elevated preoperative levels of CRP and AFP are significantly associated with the more aggressive biological tumor behavior and inferior prognosis following hepatectomy. Additionally, a key finding of this research is the development of the PACE risk score for the first time, combining preoperative CRP and AFP levels for patients undergoing hepatectomy for HCC. Notably, subgroup analysis in the BCLC A and B stages cohorts further illustrated the notable clinical relevance of the PACE risk score in guiding prognosis. The PACE risk score, which incorporates variables representative of the patient’s preoperative systemic inflammatory response and tumor biological behavior, may serve as a supplement to the BCLC staging system. The PACE score stands in contrast to more complex models reported in the literature, such as those based on radiomics [27, 28], genomics [29–32], and deep learning [28, 33]. While these models may exhibit superior predictive performance, their clinical adoption has been limited due to complexities in interpretation, higher costs, and extended processing times, which are less conducive to routine clinical workflows. We recognize the value of simplicity and accessibility in clinical decision-making, as endorsed by many scholars. The BCLC staging system remains in widespread use due to its user-friendly nature. The PACE score is specifically designed to complement the BCLC staging by integrating the systemic inflammatory response and tumor biology, potentially offering enhanced prognostic stratification. We emphasize its utility based on the inclusion of widely available clinical hematological markers—alpha-AFP and CRP—which are already integrated into routine clinical practice. This integration speaks to the practicability and ease of adopting the PACE score without imposing additional burdens on clinicians’ workflows.

The clinical significance of AFP in HCC has been supported by numerous studies and widely implemented in clinical practice [20, 25, 34, 35]. Compared to AFP, CRP’s prognostic value in HCC has been reported in the literature but has not been extensively studied [16–18, 36]. CRP is an effective biomarker and is associated with the progression of several malignancies [37–40]. Elevated CRP is associated with decreased survival rates in both resectable and non-resectable HCC patients [41–43]. Our multicenter, large cohort study identified the role of CRP levels as prognostic markers after hepatectomy for HCC, corroborating results from HCC research involving other curative and palliative treatments. For instance, CRP has been reported as a useful marker for assessing the efficacy of sorafenib or lenvatinib treatment [17, 44]. Rekik et al. reported that the STATE score, which incorporates CRP, can effectively predict survival after transarterial chemoembolization for intermediate HCC [43]. However, the mechanism behind the rise of CRP in HCC and its potential prognostic guidance are not fully understood. Inflammatory cytokines IL-1 and IL-6, secreted by tumor and stromal cells, could induce the rise of CRP [45]. This could further stimulate the formation of immunosuppressive immune cells. A study by Wang et al. showed a strong correlation between serum CRP levels and the infiltration of immunosuppressive myeloid cells in HCC tissue, with higher CRP levels typically associated with more infiltrating CD68 + tumor-associated macrophages and CD15 + tumor-associated neutrophils [46]. These studies may provide potential explanations for the prognostic significance of CRP in HCC, but the exact molecular mechanisms of CRP in HCC still require further elucidation.

Our study highlights the prognostic significance of the PACE risk score, derived from preoperative serum CRP and AFP levels, in HCC patients undergoing hepatectomy. Prior research has demonstrated the combined utility of AFP and CRP in HCC diagnosis and prognosis [26, 47, 48]. For instance, She et al. suggested that CRP could serve as an auxiliary marker for the HCC diagnosis, particularly AFP-negative HCC [48]. Kornberg et al. introduced a serological risk index based on AFP and CRP to predict liver transplant outcomes in advanced HCC [49]. Additionally, the CRAFITY score, based on these two markers, has been useful in determining HCC patients suitable for anti-PD-1 therapy [26, 47]. Given the above evidence, our research presents the first account of PACE risk score assisting in prognostic stratification following hepatectomy in HCC patients. A higher score suggests a more invasive tumor, poorer oncological outcomes, and more adverse recurrence patterns. This indicates the PACE score’s potential as a reliable prognostic marker for HCC patients after hepatectomy.

Our research found that the PACE risk score is a significant independent predictor for both BCLC A and B-stage cohorts. For BCLC A-stage patients, the BCLC algorithm advises radical treatment with a median OS of roughly five years [8]. In contrast, our data shows the median OS for BCLC A stage patients with low, intermediate, and high risks at 67.8, 54.6, and 24.6 months, respectively. This suggests that for BCLC A-stage patients with high PACE risk, surgical resection alone may not result in significant survival benefits, underscoring the need to consider further adjuvant or neoadjuvant therapy trials for this population. For BCLC B-stage, the standard treatment is TACE with a median OS of about 2.5 years [8]. Some researchers suggest surgical resection as an effective therapy for selected BCLC B-stage patients [50]. Our findings indicate that the PACE risk score can help identify survival subgroups post hepatectomy in BCLC B stage. Within the BCLC B stage cohort, the median OS for the low-risk, intermediate-risk, and high-risk groups are 45.3 months, 27.3 months, and 15.6 months respectively, with the latter two groups’ median OS notably less than the reported 2.5 years. As a high PACE risk score indicates tumor invasiveness, we suggest that low-risk BCLC B-stage patients may derive benefit from surgical resection, while intermediate and high-risk patients might need more integrated strategies to improve their prognosis further. However, further comparative studies are needed to confirm these findings.

Our study has several limitations. Firstly, the retrospective design of the study may be subject to selection bias and residual confounding. Secondly, the predominance of hepatitis B infections in the cohort may introduce observational bias, suggesting the need for further research conducted in other regions. Thirdly, various factors can raise CRP levels, including trauma, infection, inflammation, and tumor stimulation. We excluded patients with concomitant clinical infections and those needing emergency hepatectomy due to ruptured HCC, utilizing only the most recent CRP levels obtained within a week before surgery to minimize possible confounding. However, we accept that our data could be confounded by other factors causing CRP elevation, such as unnoticed chronic inflammatory diseases. Nevertheless, consistent with previous findings linking high CRP with poor outcomes, our data indicate a correlation between elevated CRP levels and aggressive tumor behavior, with independent prognostic relevance. Therefore, despite potential biases, the PACE risk score with CRP is useful for post-surgery prognosis in HCC patients. Last but not least, although our study underwent internal validation via bootstrap resampling, it still lacks validation from an external cohort, particularly one from a Western context with varying etiologies of liver cancer. Further research is necessary to ascertain the applicability of the PACE score in diverse populations.

In conclusion, PACE risk score serves as an effective tool for guiding postoperative risk stratification, potentially acting as a valuable supplement to the BCLC staging system. This amalgamation can offer clinicians a more precise and personalized prognostic evaluation and therapeutic suggestions, enhancing hepatectomy appropriateness assessments, postoperative management, and the effectiveness of the hepatectomy procedure.

### Electronic supplementary material

Below is the link to the electronic supplementary material.

**Supplementary Material 1: Table S1**. Baseline characteristics of patients with different baseline AFP levels and DCP levels. **Table S2**. Univariable and multivariable Cox regression analyses on risk factors of overall survival. **Table S3**. Univariable and multivariable Cox regression analyses on risk factors of recurrence-free survival. **Table S4**. Predictive performance of PACE, BCLC staging system, and PACE combined with BCLC staging system. **Table S5**. Univariable and multivariable Cox regression analyses on risk factors of overall survival in BCLC A cohort. **Table S6**. Univariable and multivariable Cox regression analyses on risk factors of recurrence-free survival in BCLC A cohort. **Table S7**. Univariable and multivariable Cox regression analyses on risk factors of overall survival in BCLC B cohort. **Table S8**. Univariable and multivariable Cox regression analyses on risk factors of recurrence-free survival in BCLC B cohort. **Figure S1**. Time-dependent area under the curve predicting recurrence-free survival (A) and overall survival (B) at various time points. **Figure S2**. Calibration curves of BCLC (A), PACE (B), BCLC combined with PACE (C) to predict 1-year, 3-year and 5-year RFS; calibration curves of BCLC (D), PACE (E), BCLC combined with PACE (F) to predict 1-year, 3-year and 5-year OS survival.

## Data Availability

All data generated or analyzed during this study are included in this article. Inquiries about further material documents can be directed to the corresponding author.

## List of abbreviations

PACE: prognostic implications of alpha-fetoprotein and c-reactive protein Elevation
AFP: alpha-fetoprotein
CRP: C-reactive protein
HBV: hepatitis B virus
HCV: hepatitis C virus
PLT: platelet
MVI: microvascular invasion
BCLC: Barcelona Clinic Liver Cancer
OS: overall survival
RFS: recurrence-free survival
