# Supplementary material for: Prognostic implications of alpha-fetoprotein and C-reactive protein elevation in hepatocellular carcinoma following resection (PACE): a large cohort study of 2770 patients
Source: BMC Cancer. 2023 Dec 5;23:1190. doi: 10.1186/s12885-023-11693-6 (PMC10696803; doi:10.1186/s12885-023-11693-6)
Supplement: Supplementary file 1 — Supplementary Material 1: Table S1. Baseline characteristics of patients with different baseline AFP levels and DCP levels. Table S2. Univariable and multivariable Cox regression analyses on risk factors of overall survival. Table S3. Univariable and multivariable Cox regression analyses on risk factors of recurrence-free survival. Table S4. Predictive performance of PACE, BCLC staging system, and PACE combined with BCLC staging system. Table S5. Univariable and multivariable Cox regression analyses on risk factors of overall survival in BCLC A cohort. Table S6. Univariable and multivariable Cox regression analyses on risk factors of recurrence-free survival in BCLC A cohort. Table S7. Univariable and multivariable Cox regression analyses on risk factors of overall survival in BCLC B cohort. Table S8. Univariable and multivariable Cox regression analyses on risk factors of recurrence-free survival in BCLC B cohort. Figure S1. Time-dependent area under the curve predicting recurrence-free survival (A) and overall survival (B) at various time points. Figure S2. Calibration curves of BCLC (A), PACE (B), BCLC combined with PACE (C) to predict 1-year, 3-year and 5-year RFS; calibration curves of BCLC (D), PACE (E), BCLC combined with PACE (F) to predict 1-year, 3-year and 5-year OS survival. [file 12885_2023_11693_MOESM1_ESM.docx]

**Prognostic Implications of Alpha-fetoprotein and C-reactive Protein Elevation in Hepatocellular Carcinoma Following Resection (PACE):**

***A Large Cohort Study of 2770 Patients***

Kong-Ying Lin *MD*^1,2*^, Qing-Jing Chen *MD*^1,2*^, Zhi-Wen Lin *MD*^1,2*^, Shi-Chuan Tang *MD*^1,2*^, Jian-Xi Zhang *MD*^3^, Si-Ming Zheng *MD*^4^, Yun-Tong Li *MD*^5^, Xian-Ming Wang *MD*^6^, Qiang Lu *MD*^7^, Jun Fu *MD*^1^, Ruo-Bin Guo *MD*^1,2^, Li-Fang Zheng *MD*^1^, Peng-Hui You *MD*^8^, Meng-Meng Wu^1^, Ke-Can Lin *MD*^1^, Wei-Ping Zhou *MD*^9^, Tian Yang *MD*^9#^, Yong-Yi Zeng *MD*^1,2,10#^

**CONTENTS**

[Table S1. Baseline characteristics of patients with different baseline AFP levels and DCP levels. 2](#_Toc150434804)

[Table S2. Univariable and multivariable Cox regression analyses on risk factors of overall survival. 5](#_Toc150434805)

[Table S3. Univariable and multivariable Cox regression analyses on risk factors of recurrence-free survival. 6](#_Toc150434806)

[Table S4. Predictive performance of PACE, BCLC staging system, and PACE combined with BCLC staging system. 7](#_Toc150434807)

[Table S5. Univariable and multivariable Cox regression analyses on risk factors of overall survival in BCLC A cohort. 8](#_Toc150434808)

[Table S6. Univariable and multivariable Cox regression analyses on risk factors of recurrence-free survival in BCLC A cohort. 9](#_Toc150434809)

[Table S7. Univariable and multivariable Cox regression analyses on risk factors of overall survival in BCLC B cohort. 10](#_Toc150434810)

[Table S8. Univariable and multivariable Cox regression analyses on risk factors of recurrence-free survival in BCLC B cohort. 11](#_Toc150434811)

[Figure S1. Time-dependent area under the curve predicting recurrence-free survival (A) and overall survival (B) at various time points. 12](#_Toc150434812)

[Figure S2. Calibration curves of BCLC (A), PACE (B), BCLC combined with PACE (C) to predict 1-year, 3-year and 5-year RFS; calibration curves of BCLC (D), PACE (E), BCLC combined with PACE (F) to predict 1-year, 3-year and 5-year OS survival. 12](#_Toc150434813)

Table S1. Baseline characteristics of patients with different baseline AFP levels and DCP levels.

| **Variables** | **AFP < 400ng/ml**  **(N=1876)** | **AFP ≥ 400ng/ml**  **(N=894)** | ***P-value*** | **CRP < 10mg/L**  **(N=2412)** | **CRP ≥ 10mg/L**  **(N=358)** | ***P-value*** |
| --- | --- | --- | --- | --- | --- | --- |
| **Age**, years, Mean (SD) | 54.5 (10.7) | 50.5 (11.5) | < 0.001 | 53.5 (11.0) | 50.8 (11.7) | < 0.001 |
| **Gender** |  |  |  |  |  |  |
| Female | 228 (12.2%) | 197 (22.0%) | < 0.001 | 387 (16.0%) | 38 (10.6%) | 0.008 |
| Male | 1648 (87.8%) | 697 (78.0%) |  | 2025 (84.0%) | 320 (89.4%) |  |
| **Diabetes** | 203 (10.8%) | 56 (6.3%) | < 0.001 | 236 (9.8%) | 23 (6.4%) | 0.042 |
| **Hypertension** | 379 (20.2%) | 146 (16.3%) | 0.015 | 454 (18.8%) | 71 (19.8%) | 0.649 |
| **Etiology** |  |  |  |  |  |  |
| HBV | 1571 (83.7%) | 787 (88.0%) | 0.013 | 2046 (84.8%) | 312 (87.2%) | 0.056 |
| HCV | 29 (1.5%) | 6 (0.7%) |  | 35 (1.5%) | 0 (0%) |  |
| Non-B, non-C | 271 (14.4%) | 99 (11.1%) |  | 325 (13.5%) | 45 (12.6%) |  |
| HBV, HCV | 5 (0.3%) | 2 (0.2%) |  | 6 (0.2%) | 1 (0.3%) |  |
| **Child-Pugh class** |  |  |  |  |  |  |
| A | 1746 (93.1%) | 818 (91.5%) | 0.141 | 2254 (93.4%) | 310 (86.6%) | < 0.001 |
| B | 130 (6.9%) | 76 (8.5%) |  | 158 (6.6%) | 48 (13.4%) |  |
| **BCLC staging system** |  |  |  |  |  |  |
| 0 | 135 (7.2%) | 24 (2.7%) | < 0.001 | 156 (6.5%) | 3 (0.8%) | < 0.001 |
| A | 1463 (78.0%) | 695 (77.7%) |  | 1884 (78.1%) | 274 (76.5%) |  |
| B | 278 (14.8%) | 175 (19.6%) |  | 372 (15.4%) | 81 (22.6%) |  |
| **Platelet**, Mean (SD), 109/L | 161 (66.6) | 178 (74.8) | < 0.001 | 159 (63.7) | 215 (88.1) | < 0.001 |
| **Total bilirubin,** Mean (SD), umol/L | 15.2 (12.1) | 14.8 (8.69) | 0.371 | 15.1 (11.3) | 15.3 (9.21) | 0.647 |
| **Albumin,** Mean (SD), g/L | 41.5 (3.91) | 41.4 (3.85) | 0.333 | 41.8 (3.79) | 39.4 (3.94) | < 0.001 |
| **AFP,** ng/mL |  |  |  |  |  |  |
| < 400 | 1876 (100%) | - | - | 1665 (69.0%) | 211 (58.9%) | < 0.001 |
| ≥ 400 | - | 894 (100%) |  | 747 (31.0%) | 147 (41.1%) |  |
| **CRP,** mg/L |  |  |  |  |  |  |
| < 10 | 1665 (88.8%) | 747 (83.6%) | < 0.001 | 2412 (100%) | - | - |
| ≥ 10 | 211 (11.2%) | 147 (16.4%) |  | - | 358 (100%) |  |
| **Tumor number** |  |  |  |  |  |  |
| Solitary | 1538 (82.0%) | 698 (78.1%) | 0.015 | 1961 (81.3%) | 275 (76.8%) | 0.045 |
| Multiple | 338 (18.0%) | 196 (21.9%) |  | 451 (18.7%) | 83 (23.2%) |  |
| **Tumor diameter**, Mean (SD), cm | 5.35 (3.51) | 7.38 (4.38) | < 0.001 | 5.45 (3.46) | 9.72 (4.76) | < 0.001 |
| **Satellite nodules** | 801 (42.7%) | 468 (52.3%) | < 0.001 | 1087 (45.1%) | 182 (50.8%) | 0.041 |
| **Tumor differentiation** |  |  |  |  |  |  |
| I / II | 298 (15.9%) | 25 (2.8%) | < 0.001 | 291 (12.1%) | 32 (8.9%) | 0.085 |
| III / IV | 1578 (84.1%) | 869 (97.2%) |  | 2121 (87.9%) | 326 (91.1%) |  |
| **MVI** | 689 (36.7%) | 435 (48.7%) | < 0.001 | 933 (38.7%) | 191 (53.4%) | < 0.001 |
| **Tumor capsule** |  |  |  |  |  |  |
| Complete | 328 (17.5%) | 86 (9.6%) | < 0.001 | 363 (15.0%) | 51 (14.2%) | 0.255 |
| Incomplete | 1212 (64.6%) | 650 (72.7%) |  | 1630 (67.6%) | 232 (64.8%) |  |
| None | 336 (17.9%) | 158 (17.7%) |  | 419 (17.4%) | 75 (20.9%) |  |
| **Liver cirrhosis** | 1182 (63.0%) | 561 (62.8%) | 0.897 | 1533 (63.6%) | 210 (58.7%) | 0.073 |
| **Extend of hepatectomy** |  |  |  |  |  |  |
| Minor | 1617 (86.2%) | 651 (72.8%) | < 0.001 | 2052 (85.1%) | 216 (60.3%) | < 0.001 |
| Major | 259 (13.8%) | 243 (27.2%) |  | 360 (14.9%) | 142 (39.7%) |  |
| **Intraoperative blood loss, mL** |  |  |  |  |  |  |
| < 800 | 1804 (96.2%) | 821 (91.8%) | < 0.001 | 2318 (96.1%) | 307 (85.8%) | < 0.001 |
| ≥ 800 | 72 (3.8%) | 73 (8.2%) |  | 94 (3.9%) | 51 (14.2%) |  |
| **Intraoperative blood transfusion** | 107 (5.7%) | 87 (9.7%) | < 0.001 | 125 (5.2%) | 69 (19.3%) | < 0.001 |
| **Hepatectomy type** |  |  |  |  |  |  |
| Non-anatomical | 1285 (68.5%) | 590 (66.0%) | 0.188 | 1639 (68.0%) | 236 (65.9%) | 0.443 |
| Anatomical | 591 (31.5%) | 304 (34.0%) |  | 773 (32.0%) | 122 (34.1%) |  |
| **Resection margin** |  |  |  |  |  |  |
| < 1cm | 1048 (55.9%) | 533 (59.6%) | 0.062 | 1359 (56.3%) | 222 (62.0%) | 0.043 |
| ≥ 1cm | 828 (44.1%) | 361 (40.4%) |  | 1053 (43.7%) | 136 (38.0%) |  |

**Abbreviations**: *AFP*, alpha-fetoprotein; *CRP*, C-reactive protein; *HBV,* hepatitis B virus; *HCV*, hepatitis C virus; *PLT*, platelet; *MVI*, microvascular invasion; *BCLC*, Barcelona Clinic Liver Cancer; *SD*, standard deviation.

# Table S2. Univariable and multivariable Cox regression analyses on risk factors of overall survival.

| Variables | HR comparison | UV HR (95% CI) | **UV *P*** | MV HR (95% CI) | **MV *P*** |
| --- | --- | --- | --- | --- | --- |
| Age | > 55 *vs.* ≤55 years | 0.867 (0.762-0.986) | 0.030 | NS | 0.965 |
| Gender | Male *vs.* female | 1.024 (0.856-1.225) | 0.795 |  |  |
| Diabetes | Present *vs.* absent | 1.061 (0.856-1.316) | 0.588 |  |  |
| Hypertension | Present *vs.* absent | 0.963 (0.814-1.140) | 0.663 |  |  |
| HBsAg | Positive *vs.* negative | 1.113 (0.922-1.343) | 0.266 |  |  |
| HCVAb | Positive *vs.* negative | 0.873 (0.494-1.544) | 0.641 |  |  |
| Child-Pugh | B *vs.* A | 1.569 (1.261-1.952) | < 0.001 | 1.344 (1.074-1.682) | 0.010 |
| Cirrhosis | Present *vs.* absent | 0.862 (0.756-0.981) | 0.025 | NS | 0.193 |
| PLT | <100 *vs.* ≥100 10^9^/L | 1.076 (0.91-1.273) | 0.389 |  |  |
| Tumor number | Multiple *vs.* solitary | 1.901 (1.646-2.196) | < 0.001 | 1.384 (1.178-1.625) | **<** 0.001 |
| Tumor diameter | ≥10 *vs.* <10 cm | 2.478 (2.140-2.868) | <0.001 | 1.583 (1.329-1.886) | **<** 0.001 |
| Tumor differentiation | III/IV *vs.* I/II | 2.279 (1.784-2.910) | < 0.001 | 1.674 (1.298-2.160) | **<** 0.001 |
| Tumor capsule | Incomplete *vs.* complete | 1.397 (1.148-1.699) | 0.001 | NS | 0.521 |
| Tumor capsule | None *vs.* complete | 1.986 (1.589-2.482) | < 0.001 | 1.591 (1.263-2.005) | **<** 0.001 |
| Satellite nodules | Presence *vs.* absence | 1.861 (1.635-2.118) | < 0.001 | 1.391 (1.201-1.611) | **<** 0.001 |
| Intraoperative blood loss | ≥800 ml *vs.* <800 ml | 2.163 (1.724-2.713) | < 0.001 | 1.327 (1.038-1.696) | 0.024 |
| Anatomical hepatectomy | Yes *vs.* no | 0.837 (0.726-0.964) | 0.014 | NS | 0.392 |
| Resection margin | ≥1cm *vs.* <1cm | 0.602 (0.525-0.689) | < 0.001 | 0.699 (0.594-0.822) | < 0.001 |
| **AFP** | ≥ 400 *vs.* < 400 ng/mL | 1.803 (1.583-2.053) | < 0.001 | 1.503 (1.311-1.723) | < 0.001 |
| **CRP** | ≥10 *vs.* < 10mg/L | 2.155 (1.837-2.528) | < 0.001 | 1.579 (1.323-1.884) | < 0.001 |

**Abbreviations:** *AFP,* alpha-fetoprotein; *CRP,* C-reactive protein; *HBV,* hepatitis B virus; *HCV*, hepatitis C virus; *PLT*, platelet; *HR*, hazard ratio; *CI*, confidence interval; UV, univariable; MV multivariable; NS, not significant. Schoenfeld residuals test for proportional hazards assumption: met (*P* = 0.139).

# Table S3. Univariable and multivariable Cox regression analyses on risk factors of recurrence-free survival.

| Variables | HR comparison | UV HR (95% CI) | **UV *P*** | MV HR (95% CI) | **MV *P*** |
| --- | --- | --- | --- | --- | --- |
| Age | > 55 *vs.* ≤55 years | 0.964 (0.863-1.076) | 0.512 |  |  |
| Gender | Male *vs.* female | 1.044 (0.895-1.218) | 0.582 |  |  |
| Diabetes | Present *vs.* absent | 0.989 (0.817-1.197) | 0.910 |  |  |
| Hypertension | Present *vs.* absent | 0.887 (0.767-1.026) | 0.107 |  |  |
| HBsAg | Positive *vs.* negative | 1.355 (1.142-1.608) | 0.001 | NS | 0.534 |
| HCVAb | Positive *vs.* negative | 0.840 (0.513-1.376) | 0.490 |  |  |
| Child-Pugh | B *vs.* A | 1.318 (1.078-1.611) | 0.007 | 1.332 (1.066-1.665) | 0.012 |
| Cirrhosis | Present *vs.* absent | 1.078 (0.961-1.210) | 0.199 |  |  |
| PLT | <100 *vs.* ≥100 10^9^/L | 0.981 (0.844-1.140) | 0.801 |  |  |
| Tumor number | Multiple *vs.* solitary | 1.742 (1.531-1.981) | < 0.001 | 1.379 (1.174-1.619) | **<0.001** |
| Tumor diameter | ≥10 *vs.* <10 cm | 2.163 (1.896-2.468) | <0.001 | 1.612 (1.355-1.917) | **<0.001** |
| Tumor differentiation | III/IV *vs.* I/II | 1.399 (1.166-1.678) | < 0.001 | 1.672 (1.296-2.157) | <0.001 |
| Tumor capsule | Incomplete *vs.* complete | 1.214 (1.035-1.424) | 0.017 | NS | 0.494 |
| Tumor capsule | None *vs.* complete | 1.428 (1.180-1.728) | < 0.001 | 1.600 (1.270-2.014) | <0.001 |
| Satellite nodules | Presence *vs.* absence | 1.396 (1.250-1.560) | < 0.001 | 1.394 (1.204-1.615) | <0.001 |
| Intraoperative blood loss | ≥800 ml *vs.* <800 ml | 1.975 (1.599-2.440) | < 0.001 | 1.324 (1.036-1.693) | 0.025 |
| **Anatomical** hepatectomy | Yes *vs.* no | 0.771 (0.682-0.872) | < 0.001 | NS | 0.449 |
| Resection margin | ≥1cm *vs.* <1cm | 0.678 (0.605-0.761) | < 0.001 | 0.697 (0.592-0.819) | **<0.001** |
| **AFP** | ≥ 400 *vs.* < 400 ng/mL | 1.539 (1.374-1.725) | < 0.001 | 1.494 (1.304-1.710) | **<0.001** |
| **CRP** | ≥10 *vs.* < 10mg/L | 1.830 (1.580-2.120) | < 0.001 | 1.570 (1.316-1.874) | **<0.001** |

**Abbreviations:** *AFP,* alpha-fetoprotein; *CRP,* C-reactive protein; *HBV,* hepatitis B virus; *HCV*, hepatitis C virus; *PLT*, platelet; *HR*, hazard ratio; *CI*, confidence interval; UV, univariable; MV multivariable; NS, not significant. Schoenfeld residuals test for proportional hazards assumption: met (P = 0.106).

# Table S4. Predictive performance of PACE, BCLC staging system, and PACE combined with BCLC staging system.

| **Performance testing** | **OS** | | | **RFS** | | |
| --- | --- | --- | --- | --- | --- | --- |
|  | **BCLC** | **PACE** | **PACE + BCLC** | **BCLC** | **PACE** | **PACE + BCLC** |
| **C-index** | 0.573 (0.544, 0.603) | 0.604 (0.569, 0.639) | 0.638 (0.601, 0.675) | 0.561 (0.535, 0.587) | 0.584 (0.554, 0.613) | 0.610 (0.579, 0.642) |
| **Time-dependent ROC** | | | | | | |
| **1-year** | 0.587 (0.560, 0.615) | 0.637 (0.604, 0.669) | 0.674 (0.641, 0.706) | 0.584 (0.566, 0.602) | 0.616 (0.594, 0.637) | 0.649 (0.627, 0.672) |
| **3-year** | 0.593 (0.573, 0.613) | 0.628 (0.605, 0.651) | 0.671 (0.646, 0.695) | 0.586 (0.568, 0.603) | 0.584 (0.562, 0.605) | 0.633 (0.610, 0.656) |
| **5-year** | 0.596 (0.568, 0.624) | 0.608 (0.577, 0.640) | 0.659 (0.624, 0.693) | 0.597 (0.567, 0.627) | 0.583 (0.547, 0.618) | 0.642 (0.604, 0.681) |

**Abbreviations:** *OS*, overall survival; *RFS*, recurrence-free survival; *C-index,* concordance index; *Time-dependent ROC,* time dependent receiver operating characteristic.

# Table S5. Univariable and multivariable Cox regression analyses on risk factors of overall survival in BCLC A cohort.

| Variables | HR comparison | UV HR (95% CI) | **UV *P*** | MV HR (95% CI) | **MV *P*** |
| --- | --- | --- | --- | --- | --- |
| Age | > 55 *vs.* ≤55 years | 0.899 (0.773-1.046) | 0.169 |  |  |
| Gender | Male *vs.* female | 0.962 (0.784-1.181) | 0.712 |  |  |
| Diabetes | Present *vs.* absent | 1.193 (0.934-1.523) | 0.157 |  |  |
| Hypertension | Present *vs.* absent | 1.030 (0.850-1.248) | 0.765 |  |  |
| HBsAg | Positive *vs.* negative | 1.189 (0.953-1.483) | 0.125 |  |  |
| HCVAb | Positive *vs.* negative | 0.686 (0.326-1.445) | 0.322 |  |  |
| Child-Pugh | B *vs.* A | 1.518 (1.171-1.968) | 0.002 | NS | 0.411 |
| Cirrhosis | Present *vs.* absent | 0.870 (0.747-1.014) | 0.075 |  |  |
| PLT | <100 *vs.* ≥100 10^9^/L | 1.115 (0.913-1.362) | 0.286 |  |  |
| Tumor number | Multiple *vs.* solitary | 0.833 (0.545-1.275) | 0.400 |  |  |
| Tumor diameter | ≥10 *vs.* <10 cm | 2.460 (2.068-2.926) | < 0.001 | 1.700 (1.386-2.084) | < 0.001 |
| Tumor differentiation | III/IV *vs.* I/II | 2.456 (1.824-3.306) | < 0.001 | 1.914 (1.405-2.608) | < 0.001 |
| Tumor capsule | Incomplete *vs.* complete | 1.416 (1.131-1.773) | 0.002 | NS | 0.328 |
| Tumor capsule | None *vs.* complete | 1.828 (1.405-2.378) | < 0.001 | 1.608 (1.225-2.112) | 0.001 |
| Satellite nodules | Presence *vs.* absence | 1.493 (1.279-1.744) | < 0.001 | 1.313 (1.118-1.541) | 0.001 |
| Intraoperative blood loss | ≥800 ml *vs.* <800 ml | 2.116 (1.606-2.788) | < 0.001 | 1.421 (1.059-1.907) | 0.019 |
| **Anatomical** hepatectomy | Yes *vs.* no | 0.845 (0.715-0.998) | 0.048 | NS | 0.699 |
| Resection margin | ≥1cm *vs.* <1cm | 0.614 (0.524-0.719) | <0.001 | 0.656 (0.543-0.794) | <0.001 |
| **PACE risk score** | Intermediate-risk *vs.* low-risk | 1.705 (1.454-1.999) | <0.001 | 1.448 (1.228-1.706) | <0.001 |
| **PACE risk score** | High-risk *vs.* low-risk | 3.715 (2.846-4.849) | <0.001 | 2.468 (1.831-3.327) | <0.001 |

**Abbreviations:** *PACE*, **P**rognostic implications of **A**lpha-fetoprotein and **C**-reactive protein **E**levation; *HBV*, hepatitis B virus; *HCV*, hepatitis C virus; *PLT*, platelet; *HR*, hazard ratio; *CI*, confidence interval; UV, univariable; MV multivariable; NS, not significant. Schoenfeld residuals test for proportional hazards assumption: met (*P* = 0.200).

# Table S6. Univariable and multivariable Cox regression analyses on risk factors of recurrence-free survival in BCLC A cohort.

| Variables | HR comparison | UV HR (95% CI) | **UV *P*** | MV HR (95% CI) | **MV *P*** |
| --- | --- | --- | --- | --- | --- |
| Age | > 55 *vs.* ≤55 years | 0.924 (0.813-1.050) | 0.224 |  |  |
| Gender | Male *vs.* female | 0.989 (0.831-1.176) | 0.900 |  |  |
| Diabetes | Present *vs.* absent | 1.021 (0.819-1.273) | 0.853 |  |  |
| Hypertension | Present *vs.* absent | 0.871 (0.737-1.029) | 0.105 |  |  |
| HBsAg | Positive *vs.* negative | 1.482 (1.215-1.808) | < 0.001 | NS | **0.193** |
| HCVAb | Positive *vs.* negative | 0.771 (0.426-1.398) | 0.392 |  |  |
| Child-Pugh | B *vs.* A | 1.336 (1.062-1.680) | 0.013 | NS | 0.417 |
| Cirrhosis | Pesent *vs.* absent | 1.109 (0.971-1.266) | 0.127 |  |  |
| PLT | <100 *vs.* ≥100 10^9^/L | 1.092 (0.918-1.298) | 0.320 |  |  |
| Tumor number | Multiple *vs.* solitary | 0.969 (0.694-1.352) | 0.853 |  |  |
| Tumor diameter | ≥10 *vs.* <10 cm | 2.088 (1.789-2.436) | <0.001 | 1.714 (1.397-2.102) | **< 0.001** |
| Tumor differentiation | III/IV *vs.* I/II | 1.515 (1.222-1.880) | < 0.001 | 1.904 (1.398-2.594) | **< 0.001** |
| Tumor capsule | Incomplete *vs.* complete | 1.217 (1.017-1.457) | 0.032 | NS | 0.325 |
| Tumor capsule | None *vs.* Complete | 1.320 (1.056-1.650) | 0.015 | 1.622 (1.235-2.131) | **0.001** |
| Satellite nodules | Presence *vs.* absence | 1.258 (1.103-1.434) | 0.001 | 1.312 (1.118-1.540) | **0.001** |
| Intraoperative blood loss | ≥800 ml *vs.* <800 ml | 1.902 (1.478-2.447) | < 0.001 | 1.426 (1.063-1.914) | 0.018 |
| **Anatomical** hepatectomy | yes *vs.* no | 0.807 (0.701-0.930) | 0.003 | NS | 0.721 |
| Resection margin | ≥1cm *vs.* <1cm | 0.707 (0.620-0.806) | < 0.001 | 0.655 (0.541-0.792) | **< 0.001** |
| **PACE risk score** | Intermediate-risk *vs.* low-risk | 1.394 (1.218-1.594) | < 0.001 | 1.432 (1.214-1.689) | **< 0.001** |
| **PACE risk score** | High-risk *vs.* low-risk | 2.679 (2.089-3.435) | < 0.001 | 2.424 (1.796-3.271) | **< 0.001** |

**Abbreviations:** *PACE*, **P**rognostic implications of **A**lpha-fetoprotein and **C**-reactive protein **E**levation; *HBV*, hepatitis B virus; *HCV*, hepatitis C virus; *PLT*, platelet; *HR*, hazard ratio; *CI*, confidence interval; UV, univariable; MV multivariable; NS, not significant. Schoenfeld residuals test for proportional hazards assumption: met (*P* = 0.152).

.

# Table S7. Univariable and multivariable Cox regression analyses on risk factors of overall survival in BCLC B cohort.

| Variables | HR comparison | UV HR (95% CI) | **UV *P*** | MV HR (95% CI) | **MV *P*** |
| --- | --- | --- | --- | --- | --- |
| Age | > 55 *vs.* ≤55 years | 0.592 (0.453-0.774) | <0.001 | 0.728 (0.551-0.962) | 0.026 |
| Gender | Male *vs.* female | 0.970 (0.656-1.433) | 0.878 |  |  |
| Diabetes | Present *vs.* absent | 0.685 (0.418-1.122) | 0.133 |  |  |
| Hypertension | Present *vs.* absent | 0.734 (0.500-1.078) | 0.115 |  |  |
| HBsAg | Positive *vs.* negative | 1.008 (0.686-1.480) | 0.968 |  |  |
| HCVAb | Positive *vs.* negative | 1.671 (0.687-4.065) | 0.257 |  |  |
| Child-Pugh | B *vs.* A | 1.490 (0.959-2.315) | 0.076 |  |  |
| Cirrhosis | Present *vs.* absent | 0.918 (0.706-1.196) | 0.527 |  |  |
| PLT | <100 *vs.* ≥100 10^9^/L | 1.149 (0.811-1.627) | 0.435 |  |  |
| Tumor number | ≥ 3 vs 2 | 1.512 (1.167-1.959) | 0.002 | NS | 0.160 |
| Tumor diameter | ≥10 *vs.* <10 cm | 1.732 (1.310-2.291) | <0.001 | NS | 0.521 |
| Tumor differentiation | III/IV *vs.* I/II | 1.697 (0.986-2.924) | 0.056 |  |  |
| Tumor capsule | Incomplete *vs.* complete | 0.947 (0.603-1.486) | 0.812 | NS | 0.258 |
| Tumor capsule | None *vs.* complete | 1.708 (1.054-2.769) | 0.03 | NS | 0.264 |
| Satellite nodules | Presence *vs.* absence | 2.184 (1.448-3.292) | < 0.001 | 1.780 (1.163-2.725) | 0.008 |
| Intraoperative blood loss | ≥800 ml *vs.* <800 ml | 1.649 (1.096-2.481) | 0.016 | NS | 0.680 |
| **Anatomical** hepatectomy | Yes *vs.* no | 0.828 (0.613-1.119) | 0.219 |  |  |
| Resection margin | ≥1cm *vs.* <1cm | 0.688 (0.510-0.928) | 0.014 | NS | 0.127 |
| **PACE risk score** | Intermediate-risk *vs.* low-risk | 1.439 (1.0919-1.899) | 0.010 | 1.312 (0.974-1.766) | 0.074 |
| **PACE risk score** | High-risk *vs.* low-risk | 3.205 (2.139-4.803) | < 0.001 | 2.680 (1.675-4.289) | < 0.001 |

**Abbreviations:** *PACE*, **P**rognostic implications of **A**lpha-fetoprotein and **C**-reactive protein **E**levation; *HBV*, hepatitis B virus; *HCV*, hepatitis C virus; *PLT*, platelet; *HR*, hazard ratio; *CI*, confidence interval; UV, univariable; MV multivariable; NS, not significant. Schoenfeld residuals test for proportional hazards assumption: met (*P* = 0.175).

# Table S8. Univariable and multivariable Cox regression analyses on risk factors of recurrence-free survival in BCLC B cohort.

| Variables | HR comparison | UV HR (95% CI) | **UV *P*** | MV HR (95% CI) | **MV *P*** |
| --- | --- | --- | --- | --- | --- |
| Age | > 55 *vs.* ≤55 years | 0.910 (0.716-1.157) | 0.443 |  |  |
| Gender | Male *vs.* female | 0.968 (0.669-1.403) | 0.865 |  |  |
| Diabetes | Present *vs.* absent | 0.973 (0.649-1.458) | 0.894 |  |  |
| Hypertension | Present *vs.* absent | 0.980 (0.709-1.354) | 0.901 |  |  |
| HBsAg | Positive *vs.* negative | 0.923 (0.646-1.317) | 0.658 |  |  |
| HCVAb | Positive *vs.* negative | 0.831 (0.309-2.231) | 0.713 |  |  |
| Child-Pugh | B *vs.* A | 0.930 (0.576-1.502) | 0.766 |  |  |
| Cirrhosis | Present *vs.* absent | 0.989 (0.773-1.265) | 0.930 |  |  |
| PLT | <100 *vs.* ≥100 10^9^/L | 0.740 (0.512-1.070) | 0.110 |  |  |
| Tumor number | ≥ 3 vs 2 | 1.499 (1.179-1.908) | 0.001 | 1.472 (1.131-1.916) | 0.004 |
| Tumor diameter | ≥10 *vs.* <10 cm | 1.758 (1.352-2.285) | <0.001 | NS | 0.111 |
| Tumor differentiation | III/IV *vs.* I/II | 0.887 (0.583-1.349) | 0.575 |  |  |
| Tumor capsule | Incomplete *vs.* complete | 1.027 (0.680-1.552) | 0.898 |  |  |
| Tumor capsule | None *vs.* complete | 1.243 (0.791-1.952) | 0.346 |  |  |
| Satellite nodules | Presence *vs.* absence | 0.938 (0.693-1.270) | 0.679 |  |  |
| Intraoperative blood loss | ≥800 ml *vs.* <800 ml | 1.674 (1.123-2.495) | 0.011 | NS | 0.717 |
| **Anatomical** hepatectomy | Yes *vs.* no | 0.790 (0.600-1.042) | 0.095 |  |  |
| Resection margin | ≥1cm *vs.* <1cm | 0.894 (0.688-1.162) | 0.402 |  |  |
| **PACE risk score** | Intermediate-risk *vs.* low-risk | 1.570 (1.217-2.024) | 0.001 | 1.331 (0.994-1.782) | 0.055 |
| **PACE risk score** | High-risk *vs.* low-risk | 3.474 (2.338-5.164) | < 0.001 | 2.891 (1.815-4.605) | <0.001 |

**Abbreviations:** *PACE*, **P**rognostic implications of **A**lpha-fetoprotein and **C**-reactive protein **E**levation; *HBV*, hepatitis B virus; *HCV*, hepatitis C virus; *PLT*, platelet; *HR*, hazard ratio; *CI*, confidence interval; UV, univariable; MV multivariable; NS, not significant. Schoenfeld residuals test for proportional hazards assumption: met (P = 0.220).

# Figure S1. Time-dependent area under the curve predicting recurrence-free survival (A) and overall survival (B) at various time points.

**
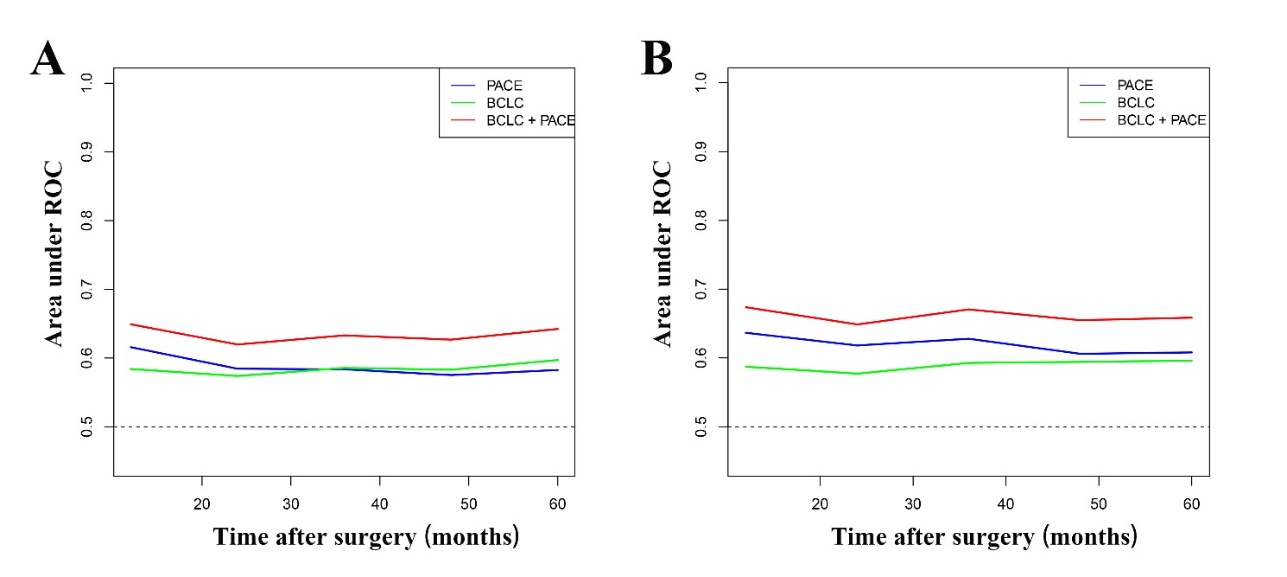
**

**Abbreviations:** *PACE*, **P**rognostic implications of **A**lpha-fetoprotein and **C**-reactive protein **E**levation; *BCLC*, Barcelona Clinic Liver Cancer.

# Figure S2. Calibration curves of BCLC (A), PACE (B), BCLC combined with PACE (C) to predict 1-year, 3-year and 5-year RFS; calibration curves of BCLC (D), PACE (E), BCLC combined with PACE (F) to predict 1-year, 3-year and 5-year OS survival.


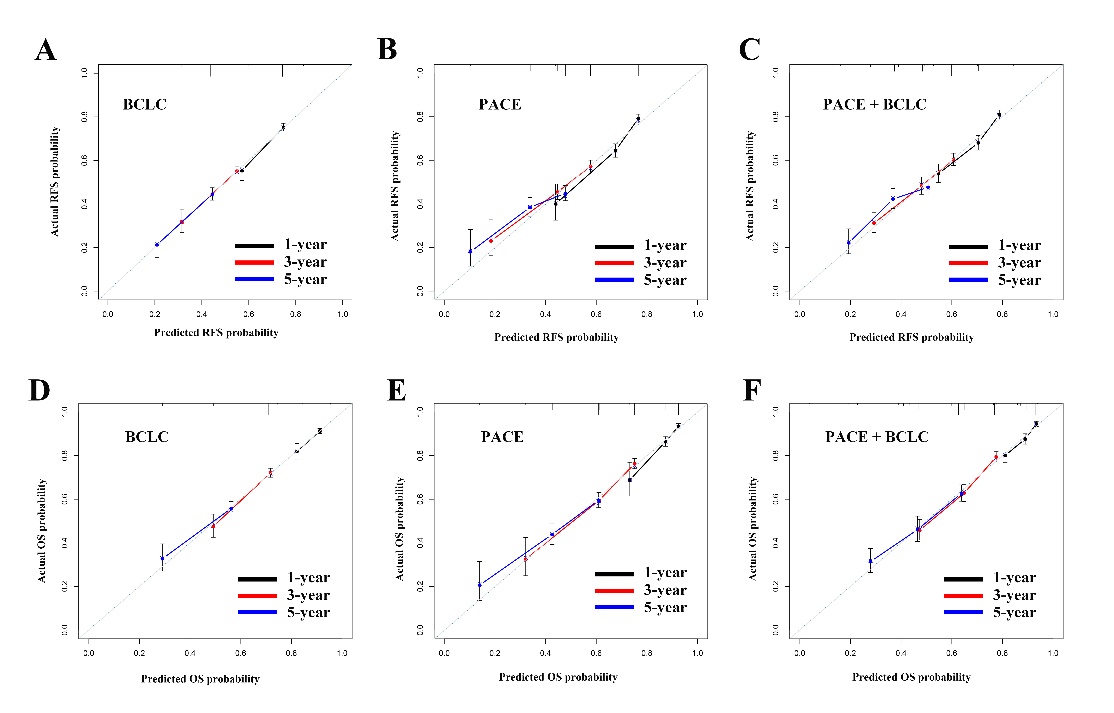


**Abbreviations:** *PACE*, **P**rognostic implications of **A**lpha-fetoprotein and **C**-reactive protein **E**levation; *BCLC*, Barcelona Clinic Liver Cancer.
